# Asynchronous Response of Tropical Forest Leaf Phenology to Seasonal and El Niño-Driven Drought

**DOI:** 10.1371/journal.pone.0011325

**Published:** 2010-06-25

**Authors:** Stephanie Pau, Gregory S. Okin, Thomas W. Gillespie

**Affiliations:** Department of Geography, University of California Los Angeles, Los Angeles, California, United States of America; Centre National de la Recherche Scientifique, France

## Abstract

The Hawaiian Islands are an ideal location to study the response of tropical forests to climate variability because of their extreme isolation in the middle of the Pacific, which makes them especially sensitive to El Niño-Southern Oscillation (ENSO). Most research examining the response of tropical forests to drought or El Niño have focused on rainforests, however, tropical dry forests cover a large area of the tropics and may respond very differently than rainforests. We use satellite-derived Normalized Difference Vegetation Index (NDVI) from February 2000-February 2009 to show that rainforests and dry forests in the Hawaiian Islands exhibit asynchronous responses in leaf phenology to seasonal and El Niño-driven drought. Dry forest NDVI was more tightly coupled with precipitation compared to rainforest NDVI. Rainforest cloud frequency was negatively correlated with the degree of asynchronicity (Δ_NDVI_) between forest types, most strongly at a 1-month lag. Rainforest green-up and dry forest brown-down was particularly apparent during the 2002–003 El Niño. The spatial pattern of NDVI response to the NINO 3.4 Sea Surface Temperature (SST) index during 2002–2003 showed that the leeward side exhibited significant negative correlations to increased SSTs, whereas the windward side exhibited significant positive correlations to increased SSTs, most evident at an 8 to 9-month lag. This study demonstrates that different tropical forest types exhibit asynchronous responses to seasonal and El Niño-driven drought, and suggests that mechanisms controlling dry forest leaf phenology are related to water-limitation, whereas rainforests are more light-limited.

## Introduction

The response of terrestrial ecosystems to climate variability such as El Niño-Southern Oscillation (ENSO) serves as an important analogy for ecosystem responses to projected future climate change. Tropical ecosystems are particularly important for understanding the mechanisms of vegetation response to climate change because they are undergoing rapid change [1] and play a unique role in the global carbon cycle [2]. One study has shown that net primary production has been increasing in recent years with the largest increase occurring in tropical ecosystems, most likely due to declining cloud cover and associated increase in solar radiation [3].

Most research examining the response of tropical forests to drought or El Niño have focused on rainforests in the Amazon [4], [5], [6], [7], Panama [8], [9], [10] or Southeast Asia [11], [12], even though tropical dry forests cover a large area of the tropics and may respond very differently compared to rainforests. One of the few studies that included different forest types was by Condit et al. [10]. They assessed the growth and mortality of tree species across a precipitation gradient in a Panamanian tropical forest. Contrary to the response of Southeast Asian forests, mortality was not elevated at wet and intermediate forests during the 1998 El Niño drought, but was at the dry forest site. Instead, tree growth was observed to be higher during 1998 and this was hypothesized to be related to increased photosynthetically active radiation (PAR) during the 1998 El Niño, consistent with the relationship between light and forest phenology proposed by Wright and van Schaik [13].

Recent controversy over satellite-observed dry season green-up in Amazon forests has highlighted the lack of understanding of tropical forest productivity and their response to drought, ENSO, and projected future climate change [14]. The difficulty in interpreting forest response to drought in the Amazon may be attributed to different methods in processing the satellite data [14] and apparent inconsistencies between satellite-observed green-up [4], [5] and plot-based evidence of mortality and biomass loss [7]. The Amazon basin is subject to large fires, contributing to increased aerosols, which may confound the effect of cloud cover on incoming solar radiation [15]. Additionally, the hydrologic cycle of the Amazon basin has been shown to be unique, recycling a significant portion of its own precipitation from evaporation in the same basin [16]. Thus changes in solar radiation attributed to cloud cover may be unique to the Amazon basin. Previous studies have hypothesized that an increase in cloud cover during the wet season explained a decline in productivity, but did not include cloud data. For these reasons it remains unclear whether a reduction in clouds during a drought results in increased productivity, and if this mechanism is specific to the large expanses of Amazonian forests.

The Hawaiian Islands are an ideal location to study the response of tropical forests to climate variations because of their extreme isolation in the middle of the Pacific, which makes them unique as signals for climate variability [17]. The greatest source of interannual climate variability in the Hawaiian Islands is ENSO [18], [19], [20], [21]. El Niños result in drought-like conditions for the Hawaiian Islands and increased precipitation during La Niña, in part because they receive a majority of their precipitation from the northeast trade winds, but also because they are located in the subsidence zone of the Hadley cell [18], [21]. Greater subsidence is expected to correspond to a reduction in cloud cover during El Niño events. Both tropical rainforests and tropical dry forests occur in Hawaii and these forests offer clear signals of forest phenology and productivity response to tropical Pacific climate variations such as ENSO. The Normalized Difference Vegetation Index (NDVI) from satellite data is capable of measuring photosynthetic activity and seasonal to interannual changes in leaf cover as measures of forest leaf phenology. NDVI is a ratio between the red and infrared spectral regions of the electromagnetic spectrum and has been shown both theoretically and experimentally to be associated with photosynthetic activity [22], [23], [24] and aboveground net primary productivity [25], [26], [27]. In this paper we examine the NDVI of rainforests and dry forests and examine its relationship to precipitation and cloud cover during seasonal and El Niño-driven drought. We test the hypothesis that rainforests and dry forests exhibit different responses to drought and that this difference is attributed to a reduction in clouds over rainforests.

## Methods

### Satellite data

**Figure 1:**
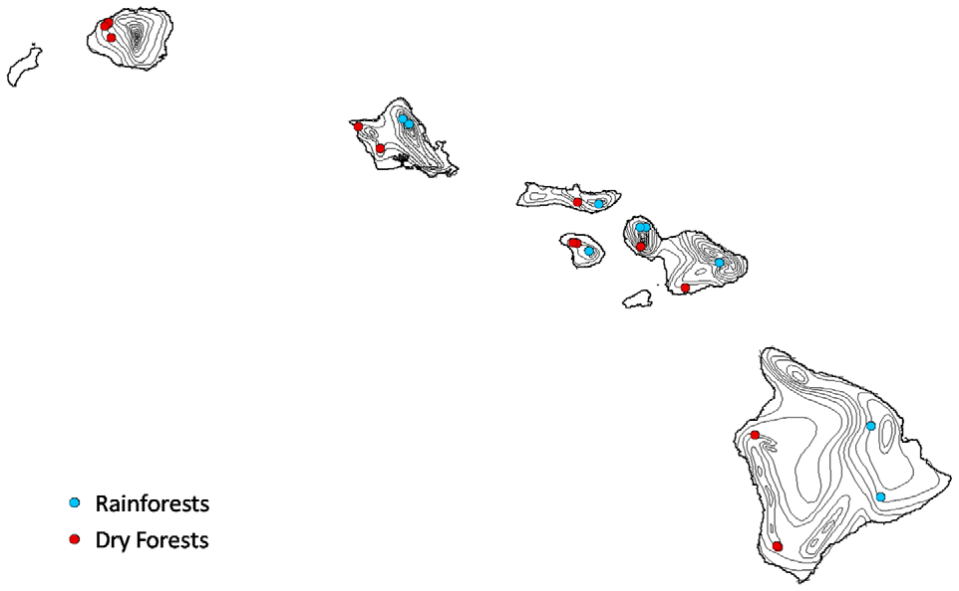
Study sites. Nine rainforest (blue) and fifteen dry forest (red) sites across six of the main Hawaiian Islands with precipitation isohyets (black lines).

Terra MODIS (Moderate Resolution Imaging Spectroradiometer) NDVI from the Vegetation Indices (VI) 16-day 250-m product (MOD13Q1) Collection 5 was used to assess leaf phenology from February 2000 - February 2009. Pixel quality filtering conformed exactly to Samanta et al. [14], who have challenged methods in other satellite studies. Valid pixels had a “MODLAND_QA” flag equal to 0 (“good quality”) or 1 (“check other QA”). Checking “other QA” included filtering out data with clouds (adjacent cloud, mixed clouds, and possible shadow), aerosols (high and climatology aerosols), and possible shadow. Pixels centered over fifteen dry forest sites, which occur within regions where potential evapotranspiration (PET) exceeds mean annual precipitation (MAP) as defined by Price et al. [28], were averaged for a representative dry forest phenology time-series (Figure 1). Dry forest sites were selected based on their dominance of native species, location in a managed and protected area versus private land, and closed-canopy cover. Pixels centered over nine rainforest sites were averaged for a representative rainforest phenology. Rainforest sites were selected from the 2008 Hawaii GAP Analysis Program (HI-GAP) vegetation data from locations that were native species-dominated, closed-canopy, and with at least 30% of the NDVI data passing the VI quality threshold above.

Cloud frequency over each rainforest pixel was identified using the “Clear_sky_days” layer in MODIS Land Surface Temperature (LST) 8-day 1-km product (MOD11A2) Collection 5. This layer is defined using the MODIS Cloud Mask product (MOD35_L2) algorithm, which is based on a combination of multiple visible and infrared threshold and consistency tests including spectral regions not used for NDVI. Clear-sky pixels at a confidence of ≥95% over land ≤2000 m and ≥66% over land >2000 m, plus an examination of 32-day temporal variations, are provided for each day in the 8-day LST dataset [29]. Days that did not pass the binary (yes/no) clear-sky test (“Clear Sky” flag = 1) were totaled for each 8-day period at each rainforest site, averaged over all rainforest sites, and then averaged by month, which is referred to as cloud frequency.

### Precipitation and SST data

Hydronet archived precipitation data from NWS NOAA were averaged into monthly totals from original 15-minute data. Representative leeward (dry side of islands) precipitation was created from an average of fourteen stations on the leeward sides of Oahu, Molokai, Maui, Lanai, and Big Island, and representative windward (wet side of islands) precipitation was an average of eight stations on the windward sides of Kauai, Oahu, Maui, and Big Island. Although the Hydronet archive includes more stations, some had considerable data quality issues such as missing data for an entire month, therefore not all Hydronet stations were included in the analyses. Leeward stations and windward stations were all significantly (*P*≤0.05) and positively associated within each group. The NOAA National Centers for Environmental Prediction (NCEP) Optimum Interpolation (OI) version 2 (v2) NINO 1.2, 3, 3.4, and 4 Sea Surface Temperature (SST) indices (generated using in situ and satellite SSTs as well as SSTs simulated by sea ice cover) were used to assess El Niño or La Niña conditions. Pearson correlations were performed to determine the association between SST and precipitation during ENSO events.

### Data analysis

NDVI anomalies were calculated by subtracting each 16-day composite value from the 9-year mean (February 2000 – February 2009) of that day and then anomalies were averaged into monthly values. Precipitation anomalies were calculated by subtracting monthly averages from the 9-year mean. The annual mean, minimum, maximum, and minimum subtracted from maximum were calculated for NDVI and precipitation anomalies, as well as annual cumulative precipitation totals. Spearman rank correlation tests were used to assess the relationship between rainforest and dry forest NDVI and precipitation for each year. The degree of asynchronicity in rainforest and dry forest NDVI was identified by subtracting dry forest NDVI from rainforest NDVI using 3-month moving averages, which we have defined as Δ_NDVI_. Thus, the greater the difference between dry forest and rainforest NDVI, the greater the value of Δ_NDVI_. Pearson product moment correlations were calculated between Δ_NDVI_ and rainforest cloud frequency. Maps of Spearman rank correlations were produced on a pixel-by-pixel basis between monthly NDVI anomalies and NINO 3.4 SST index during the 2002–2003 El Niño.

## Results

**Figure 2:**
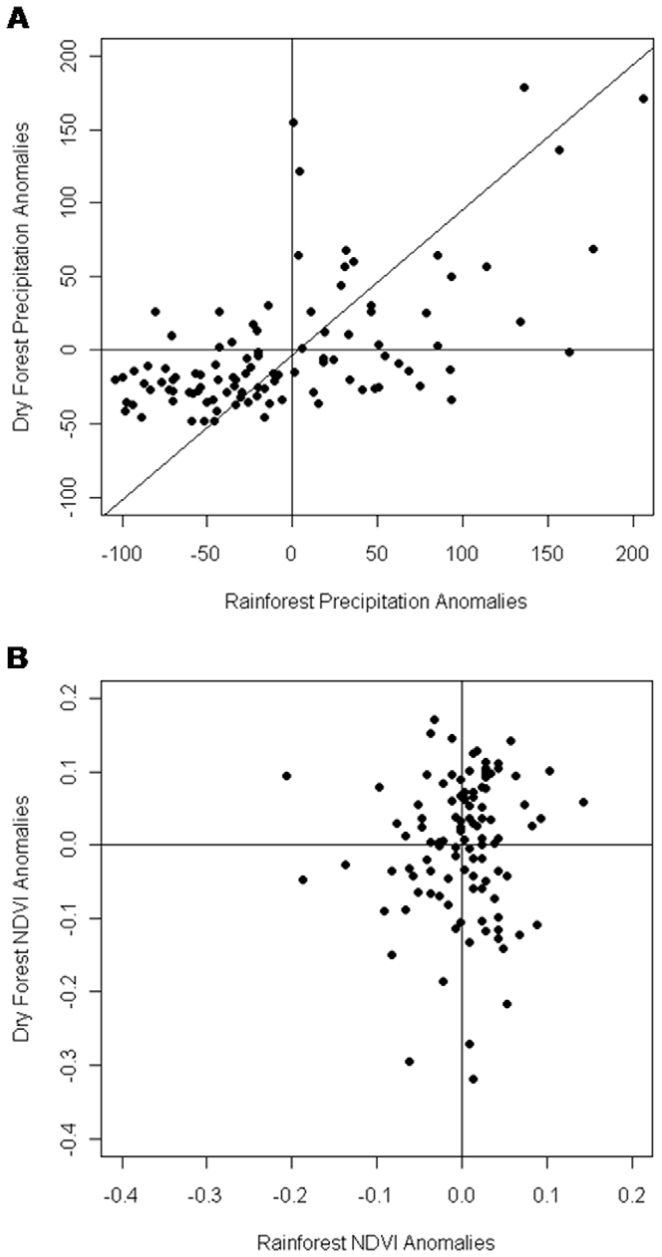
Comparison of relationship between rainforest and dry forest precipitation with rainforest and dry forest NDVI. Relationship between windward and leeward precipitation anomalies from 9-year mean (*P*<0.001, *r* = 0.566, *df* = 107) (a). Rainforest and dry forest NDVI anomalies show no relationship (*P* = 0.475, *r* = 0.070, *df* = 104) (b).

**Figure 3:**
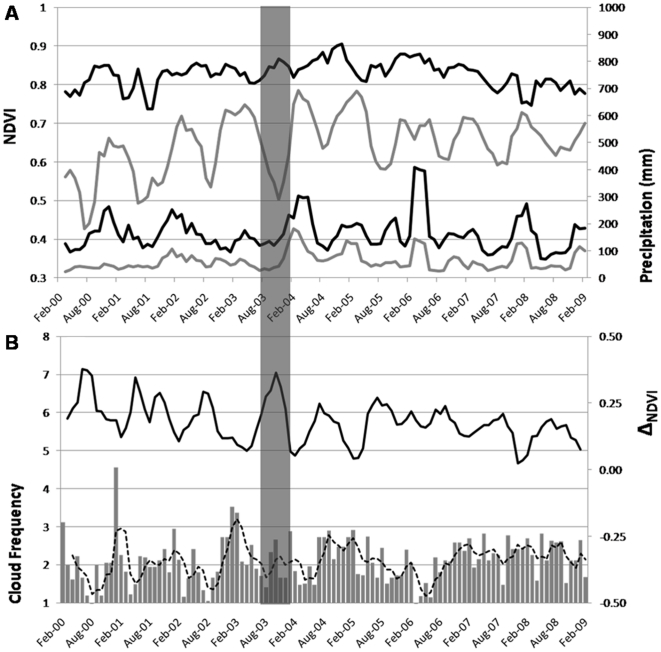
Asynchronous rainforest and dry forest NDVI and relationship to rainforest cloud frequency. Rainforest NDVI (black line) and dry forest NDVI (grey line) showing 9-year leaf phenology on left axis, and windward precipitation (black line) and leeward precipitation (grey line) on right axis. Data shown are 3-month moving averages, while analyses were performed on monthly data (a). Average cloud frequency over rainforest sites for each 8-day period (grey bars) with 3-month moving average (black dotted line) on left axis and Δ_NDVI_ (black line) on right axis (b). Grey bar highlighting 2002–2003 El Niño-driven drought.

Windward and leeward precipitation were generally similar (*P*<0.001, *r* = 0.566, *df* = 107) with more total precipitation on the windward side and higher positive anomalies compared to the leeward side (Figure 2a and 3a). However, rainforest and dry forest NDVI showed no relationship (*P* = 0.475, *r* = 0.070, *df* = 104) (Figure 2b and 3a). Dry forest NDVI was more tightly coupled to precipitation than rainforest NDVI as indicated by Spearman rank correlations (Tables 1 and 2). The strongest relationship between precipitation and rainforest NDVI was between the yearly cumulative precipitation and mean NDVI, whereas precipitation minimum and maximum were not significantly correlated to any measure of rainforest NDVI. Dry forest mean NDVI was strongly influenced by both cumulative precipitation and mean precipitation, and precipitation maximum was positively correlated to NDVI minimum, mean, and the difference between maximum and minimum NDVI for each year.

**Table 1:** Spearman rank correlations between rainforest NDVI and windward precipitation anomalies (N = 9, (*) two-tailed p<0.05, (**) p<0.01).

	NDVI max	NDVI mean	NDVI max-min	Precip min	Precip max	Precip mean	Precip cumulative
NDVI min	0.795(*)	0.895(**)	−0.285	0.377	0.209	0.536	0.669(*)
NDVI max		0.765(*)	0.250	0.200	0.233	0.667(*)	0.833(**)
NDVI mean			−0.168	0.387	0.529	0.748(*)	0.849(**)
NDVI max-min				−0.100	0.217	0.167	0.167
Precip min					0.300	−0.100	0.200
Precip max						0.467	0.650
Precip mean							0.750(*)

**Table 2:** Spearman rank correlations between dry forest NDVI and precipitation anomalies (N = 9, (*) two-tailed p<0.05, (**) p<0.01).

	NDVI max	NDVI mean	NDVI max-min	Precip min	Precip max	Precip mean	Precip cumulative
NDVI min	0.517	0.853(**)	0.833(**)	0.450	0.683(*)	0.617	0.617
NDVI max		0.766(*)	−0.167	0.133	0.433	0.783(*)	0.783(*)
NDVI mean			−0.644	0.566	0.792(*)	0.870(**)	0.870(**)
NDVI max-min				−0.500	0.800(**)	−0.550	−0.550
Precip min					0.533	0.400	0.400
Precip max						0.883(**)	0.883(**)
Precip mean							1.000(**)

During dry seasons, dry forest NDVI showed decreasing greenness while rainforest NDVI showed increasing greenness (Figure 3a). Δ_NDVI_ was negatively correlated with cloud frequency, most strongly at a 1-month lag (*P*<0.01, *rho* = −0.35, *df* = 104), but also significant at no lag (*P*<0.01, *rho* = −0.32, *df* = 104) and a 2-month lag (*P*<0.01, *rho* = −0.31, *df* = 103). Thus a decrease in the frequency of clouds corresponded to a greater degree of divergence between rainforest and dry forest NDVI (Figure 3b).

**Figure 4:**
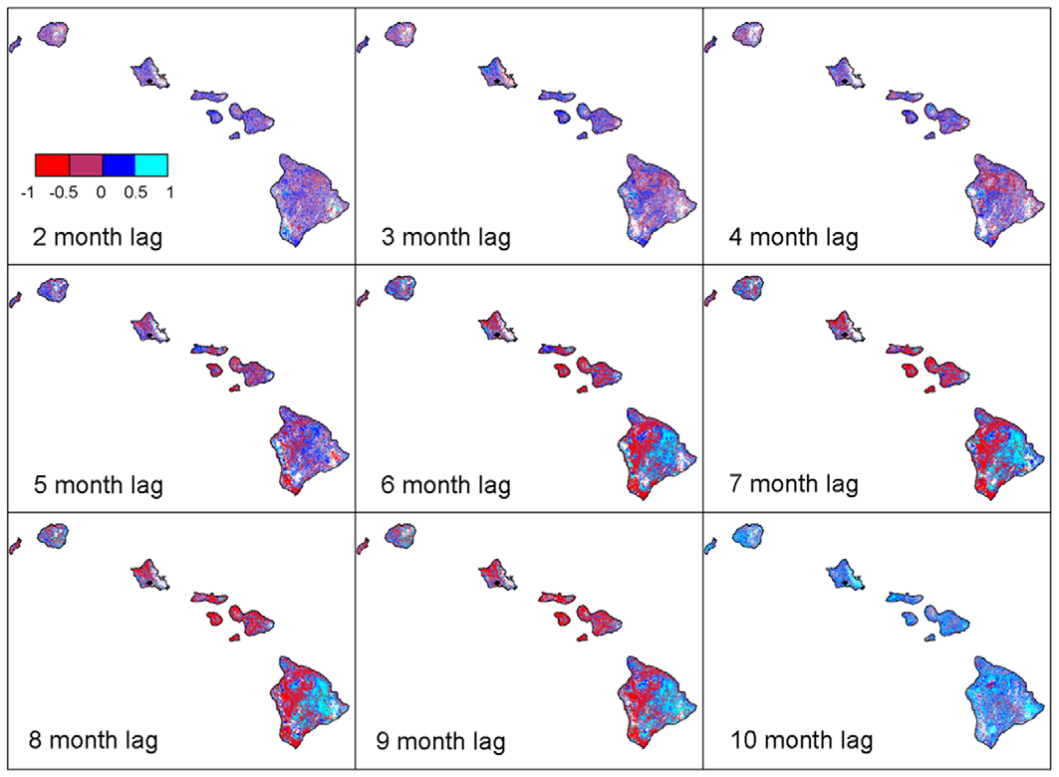
Spatial and temporal patterns in NDVI response to El Niño-driven drought. Spearman rank correlation coefficients between NDVI anomalies from 9-year mean (2000–2008) and NINO 3.4 SST index, showing strongest leeward drought response (negative correlation) and windward green-up at an 8–9-month lag during 2002–2003 El Niño event (white regions are not statistically significant). (Precipitation isohyets on Fig. 1.)

The dry season of 2003 exhibited very clear asynchronous phenology coinciding with a year of very low precipitation and low cloud frequency (Figure 3a and 3b). During the period observed, the 2002–2003 El Niño was the strongest ENSO event and the only ENSO event where NINO indices (1.2., 3, 3.4, and 4) were significantly correlated with precipitation at an 8-month lag, although the NINO 3.4 was most strongly associated (*P*<0.05, *rho* = −0.55, *df* = 22). This indicates that higher than normal SSTs were correlated with low precipitation. Asynchronous phenology was also evident in the spatial pattern of NDVI response to the NINO 3.4 SST index during the 2002–2003 El Niño (Figure 4). These maps show the progression of NDVI response to an El Niño-driven drought across the main eight Hawaiian Islands. The divergent leeward-windward response began to emerge at a 5-month lag and was strongest at an 8–9-month lag. While dry forests on the leeward side of the islands were negatively related to SSTs, showing a brown-down during drought, rainforests on the windward side of the islands exhibited positive NDVI responses to drought, or a green-up.

## Discussion

We have identified asynchronous rainforest and dry forest responses in leaf phenology to seasonal and El Niño-driven drought. Although both rainforest and dry forest regions experienced reduced precipitation during the 2002–2003 El Niño, only dry forests exhibited a brown-down response. Rainforests exhibited a green-up and this coincided with a reduction in cloud frequency and greater asynchronous leaf phenology (Δ_NDVI_). Berlin et al. [30] observed that certain cloud forest tree species on Maui showed peaked leafing during the dry season, which coincided with peaks in temperature and solar irradiance. In Neotropical rainforests experimental tests of light limitation from cloud cover [9], plot-based inventories on tree growth and mortality [10], and flux tower evidence of higher incident radiation during dry periods [31], [32] provide supporting evidence that a reduction in cloud cover can increase incoming solar radiation, which results in enhanced forest productivity during dry periods. Our results suggest a similar explanation for Hawaiian rainforest green-up during drought.

An alternative explanation for dry season green-up in Hawaiian rainforests is related to nutrient availability, which has been shown to decline at high levels of precipitation [33]. Results from a precipitation gradient on Maui demonstrated a reduction in net primary productivity (NPP) in very wet regions (>3500–4000 mm/yr) consistent with a decline in nutrient cycling, particularly nitrogen availability [33]. Additionally, the decline of epiphytes in cloud forests was observed during the 1982–1983 El Niño on Maui [17], which could result in a green-up during drought if epiphytes covering green leaves dampened spectral greenness. In spite of these alternative hypotheses, our results show a consistent NDVI pattern related to clouds. We do not argue that rainforests are drought insensitive but reveal a more complicated process of both light and water limitations on photosynthesis. For example, the very dry summers of 2007 and 2008 show lower rainforest NDVI values relative to the 9-year mean, and importantly there is no corresponding decline in clouds during this period.

Understanding of tropical dry forest response to drought is consistent with the browning-down observed in the satellite NDVI. Neotropical dry forests are known to respond to water limitations during drought [34], [10]. There have been few studies of Hawaiian dry forest phenology, but Stratton et al. [35], [36] showed that many dry forest trees obtain water from shallow soils and thus are more vulnerable to drought. Van Riper III [37] described species-specific differences in dryland species on Mauna Kea, Big Island with some species showing the greatest canopy density during the dry summer months, while other species were more tightly coupled with precipitation.

Reconciling inconsistencies between satellite observations, flux tower measurements, and plot-based surveys will require further understanding of what spectral greenness indicates in terms of carbon storage and plant growth. One important difference may be that measurements of woody biomass are capturing a slower turnover pool compared to leaves and litter, which is a faster turnover pool [38], [32]. However, equally as important is attention to differences in species composition, species-specific traits, species diversity, and forest type. Condit et al. [39], [10] have identified important differences in the drought response of forest species between size classes (saplings vs. large trees), life history (light-gap specialists vs. generalists), and forest types (dry vs. wet forests) that impede a generalized understanding of tropical forest response to drought. Huete et al. [4] contrasted responses from pastures, which exhibited dry season declines in EVI (Enhanced Vegetation Index), to intact forests showing dry season boosts in EVI. They suggested that the removal of deep-rooted trees in land converted to pasture reduced access to deep soil water. However, in Hawaii large regions showing a brown-down response to El Niño are forests and shrublands, as well as grasslands and pastures. Asynchronous response was related to the wet-dry habitat transition, not forest-grassland transition. Nonetheless it remains unclear what the roles of species-specific traits, demography, life history, and other biological factors play on the asynchronous phenology of these forest types.

### Conclusions

This study demonstrates that Hawaiian rainforests and dry forests exhibit asynchronous leaf phenology during seasonal and El Niño-driven drought. During dry seasons, dry forest NDVI showed decreasing greenness while rainforest NDVI showed increasing greenness. Dry forest NDVI was more tightly coupled with precipitation compared to rainforest NDVI. The degree of asynchronicity between forest types (Δ_NDVI_) was negatively correlated with cloud frequency, most strongly at a 1-month lag. Our results suggest that a reduction in clouds over the rainforest during dry periods may have increased solar radiation resulting in a dry season green-up. Rainforest green-up and dry forest brown-down was particularly apparent during the 2002–2003 El Niño, which was a period of low precipitation and few clouds. The spatial pattern of NDVI response to the NINO 3.4 SST index showed that the leeward side exhibited significant negative correlations to increased SSTs, whereas the windward side exhibited significant positive correlations to increased SSTs, most evident at an 8 to 9-month lag. This study demonstrates that different tropical forest types exhibit asynchronous responses to seasonal and El Niño-driven drought, and suggests that mechanisms controlling dry forest leaf phenology are related to water-limitation, whereas rainforests are more light-limited.
